# Impact of Climate Change Effects on Contamination of Cereal Grains with Deoxynivalenol

**DOI:** 10.1371/journal.pone.0073602

**Published:** 2013-09-16

**Authors:** H. J. Van der Fels-Klerx, Esther D. van Asselt, Marianne S. Madsen, Jørgen E. Olesen

**Affiliations:** 1 RIKILT Wageningen UR (Wageningen University and Research Centre), Wageningen, the Netherlands; 2 Danish Meteorological Institute, Danish Climate Centre, Copenhagen, Denmark; 3 Department of Agroecology, Aarhus University, Tjele, Denmark; Kansas State University, United States of America

## Abstract

Climate change is expected to aggravate feed and food safety problems of crops; however, quantitative estimates are scarce. This study aimed to estimate impacts of climate change effects on deoxynivalenol contamination of wheat and maize grown in the Netherlands by 2040. Quantitative modelling was applied, considering both direct effects of changing climate on toxin contamination and indirect effects via shifts in crop phenology. Climate change projections for the IPCC A1B emission scenario were used for the scenario period 2031-2050 relative to the baseline period of 1975-1994. Climatic data from two different global and regional climate model combinations were used. A weather generator was applied for downscaling climate data to local conditions. Crop phenology models and prediction models for DON contamination used, each for winter wheat and grain maize. Results showed that flowering and full maturity of both wheat and maize will advance with future climate. Flowering advanced on average 5 and 11 days for wheat, and 7 and 14 days for maize (two climate model combinations). Full maturity was on average 10 and 17 days earlier for wheat, and 19 and 36 days earlier for maize. On the country level, contamination of wheat with deoxynivalenol decreased slightly, but not significantly. Variability between regions was large, and individual regions showed a significant increase in deoxynivalenol concentrations. For maize, an overall decrease in deoxynivalenol contamination was projected, which was significant for one climate model combination, but not significant for the other one. In general, results disagree with previous reported expectations of increased feed and food safety hazards under climate change. This study illustrated the relevance of using quantitative models to estimate the impacts of climate change effects on food safety, and of considering both direct and indirect effects when assessing climate change impacts on crops and related food safety hazards.

## Introduction

Climate change effects are expected to affect crop productivity and suitability of areas to grow particular crops [1-10]. Besides these effects, climate change is expected to affect plant pathogens and associated diseases [11,12]. Recently, the impact of climate change on timing of wheat anthesis and frequency of Fusarium Head Blight (FHB) has been studied in the UK. Results showed that – with projected climate in 2050 – anthesis was estimated to occur earlier in the season and FHB epidemics were projected to be more severe [12]. Apart from fungal infections, the presence of mycotoxins, produced by *Fusarium* spp., will be affected by climate change as weather conditions during critical crop developmental stages are essential for the fungi to infect the crop and to produce toxins [13-15].

Deoxynivalenol (DON) is one of the most important mycotoxins in temperate regions of the globe, such as of Europe and North America, where the toxin is mainly produced by *F* *. graminearum* and *F* *. culmorum* [16]. DON concentrations in feed and food crops must be kept below critical limits to prevent adverse effects on animal and human health [17,18]. Although the impact of projected climate change on crop productivity and land suitability as well as crop pathogens is acknowledged (e.g. [8,12]), consequences for feed and food safety hazards, like mycotoxin contamination of crops, have only marginally been addressed to date. Several reviews on this topic have been published, pointing towards increased mycotoxin contamination of cereal grains and food safety problems [13-15,19]. However, quantitative estimates are scarce. A recent study estimated climate change effects on the presence of DON in winter wheat grown in northwest Europe [20-22].

This study aimed to quantify impacts of projected climate change on DON contamination of winter wheat (*Triticum aestivum* L.) and grain maize (*Zea mays* L.) cultivated in the Netherlands by 2040. The study used a modelling approach, and considered both the expected direct effects (due to changing weather) and indirect effects (due to shifts in crop phenology) of climate change on DON contamination in these two cereal grain crops.

## Methods

### Climate change projections

This study used data of climate change projections for the year 2040, given the α1B SRES emission scenario [23]. Climate change projection data for 2031-2050 was taken to represent the climate by 2040. The α1B emission scenario represents a mid-range scenario for greenhouse gas emissions [24]. However, up to 2050, the effect of different emission scenarios on the projected climate change is rather small [23]. The reference period of 1975-1994 was defined as the baseline from which the climate change signal was calculated. Modelled climate change projections from the ENSEMBLES project were used [25]. Data from two different combinations of a global climate model (GCM) and a regional climate model (RCM) – being ECHAM5/RACMO2 (the KNMI model) and HadCM3Q0/HadRM3Q0 (the HC model) – were used. These two model combinations were selected as they spanned a large part of the range of projected temperature and precipitation change by 2040 [26]. Climate model data were extracted from the ENSEMBLES database (available at: http://ensemblesrt3.dmi.dk).

Weather generators (WGs) are capable of generating daily weather time series statistically similar to the observed weather as required by crop simulation models [27], and they have been adopted in climate change studies as a tool to downscale climate change scenarios with high temporal and spatial resolutions based on the output from the GCMs/RCMs. The LARS Weather Generator (LARS-WG) was used to generate regional daily data, calibrated against observed data for the baseline period for the region of interest [27], and adjusted with model projected changes in monthly climate statistics between the control and scenario periods using outputs of the GCM/RCMs [28]. Output of the WG includes series of 50 synthetic years of daily data on precipitation, maximum and minimum temperature and solar radiation. Hourly temperature and relative humidity – not included in LARS WG, but needed in the mycotoxin impact models (see below) - were estimated. Daily relative humidity values were added to the time series using a randomized procedure based on the GCM/RCM data [26]. Hourly values of temperature and relative humidity were estimated from the daily values assuming that daily temperature variation can be modelled by a sine curve [29-31].

### Crop phenology models

Empirical crop development models were used to estimate climate change impacts on shifts in flowering and full maturity dates of winter wheat and grain maize. Phenology models used assumed that the period until wheat flowering depends on temperature and day-length, and the period until flowering of maize depends on temperature [9]. For both crops, the period from flowering to full maturity was assumed to depend on temperature only. The base temperatures used were 5 °C for wheat and 8 °C for maize during the entire growth period. For winter wheat the calculations of crop development was started at the 1^st^ of January, assuming that varieties would have been selected that generally would have completed their vernalisation before onset of winter. The models were calibrated using a large set of field data with records of observed dates of sowing, flowering and maturity of wheat and maize grown in north, west and central Europe during 1985-2009. This calibration showed that the duration of the phenology phases - expressed in degree-days - increased with mean temperature of the site, thus accounting for adaptation in the variety choice to the prevailing local climatic conditions [9].

The crop phenology models applied are based on well-established relationships between temperature, day-length and development rates in these crops, as described in [9]. The responses to climate change are therefore highly credible. However, responses will also depend on management in terms of sowing time and specific genotypes used [10]. In this context we assumed that such adaptations would follow the overall geographical pattern of response of temperature sum demands for individual phenophases to current mean temperature conditions across northern Europe. We used a very large dataset for this calibration, and results can therefore be seen as very accurate.

### Mycotoxin impact models

Predictive models for DON in crops grown in the Netherlands were used, including a model for winter wheat [32] and a model for grain maize [33]. The descriptive model for DON in wheat includes the following variables: region in the Netherlands (north, west, south, east), wheat resistance level against *Fusarium* spp. (level ranging from 1-10, with 1 very low to 10 very high resistance), application of late fungicides against *Fusarium* spp. (0, 1, or 2 times), wheat flowering date, duration between wheat flowering and full maturity dates (in days) – referred to as grain filling period - and weather parameters related to temperature, relative humidity and rainfall in several stages of wheat development critical for fungal infection and mycotoxin production, from two weeks before wheat flowering to full maturity date. Wheat flowering date (ordinal date) was calculated as the date at which the sum of average daily temperatures since 1^st^ of January of the particular year reached 1265 °C. Full maturity date (ordinal date) was calculated as the first date at which the sum of average daily temperatures after the calculated flowering date reached 989 °C [32]. Weather parameters included total rainfall (in mm), numbers of hours with relative humidity of 80% or higher, and average hourly temperature (in ˚C), each in the period from 17 to 10 days prior to flowering date, and average hourly temperature (in ˚C) in the consecutive period from 10 days prior to flowering date, and the two periods from 10 days after flowering date to 10 days prior to full maturity date, and from 10 days prior to the full maturity date (as well as their quadratic terms in the latter two periods). Internal validation of the predictive model for DON in wheat, using an independent randomly drawn set of data, showed a correlation between model estimates and observed DON values of around 0.80 [32]. In the present study, the model was run for resistance level 7, which represents medium resistance of the wheat variety against *Fusarium* spp., and no spraying (0 times) against *Fusarium* spp.

The model that predicts DON presence in maize grown in the Netherlands [33] is a mixed mechanistic-empirical model that describes infection during silking (SILK_INF) and subsequent growth and toxin formation by *Fusarium* *graminearum* (TOX_SYNTH):

$TOX\text{ = 1308}\text{.167 + 0}\text{.925}SILK_{INF}\text{ - 12}\text{.965}TOX\text{\_}SYNTH$ (1)

Details of the model can be found in [33]. SILK_INF depends on spore dispersal through wind and rain as well as germination of the spores influenced by temperature and relative humidity. In the present study, spore dispersal by wind was set to 1 assuming that wind speed always reaches the threshold for dispersal (3.1 km/h). Simulation runs of the baseline period showed that the threshold was reached in the majority of cases, and simulations for the future scenarios showed that wind speed will increase such that 99% is above the threshold (data not shown). The dispersal factor was fitted to DON concentrations using average daily rainfall, resulting in the following model for infection during silking:

$SILK_{INF}=(R+a)*GERM_{T}*GERM_{RH}$ (2)

where *R*: average rainfall (mm/day), *a*: parameter for dispersal (*a* = 22.74), *GERM*_*T*_: germination affected by temperature, depending on the minimum, optimum and maximum temperature for fungal germination, and *GERM*_*RH*_: germination affected by relative humidity, depending on the minimum and optimum relative humidity. Fungal growth and DON formation in maize were modelled as a function of temperature and water activity of the grain. Water activity in turn is based on the moisture content of the grain, which depends on the growth stage of the maize plant. Validation of the predictive model for DON in maize, using an independent set of monitoring data, resulted in a correlation coefficient of 0.83 between model predictions for the years 2002-2007 and average values of monitoring data in these years [33].

### Model scenarios and output

The crop phenology and mycotoxin impact models were run using climate data for the baseline period and the future scenario periods, generated for each of the two GCM/RCM combinations (KNMI and HC). Besides climate data, the mycotoxin models also used the output from the phenology models, i.e. the calculated flowering and full maturity dates for each of the two crops. In total 50 model runs were performed per grid cell (50 x 50 km) in the Netherlands (total of 31 grid cells), for each of the baseline and the two future climate scenarios (KNMI and HC).

Output included a series of 50 simulation runs (representing synthetic years) of flowering date, full maturity date, and DON concentration per grid cell in the Netherlands, for each of wheat and maize, for the baseline and the two future climate scenarios. For each of these three outputs the average, minimum and maximum values were calculated per grid cell (50 runs) for both wheat and maize. Based on the average values, differences in dates of flowering and full maturity as well as DON concentrations in the future relative to the baseline scenario period were calculated, for each grid cell and for the entire country (across grid cells). Differences were tested for their significance, using a t-test with significance level 0.05, with differences in DON concentrations evaluated on log scale.

## Results

### Climate change projections

**Figure 1:**
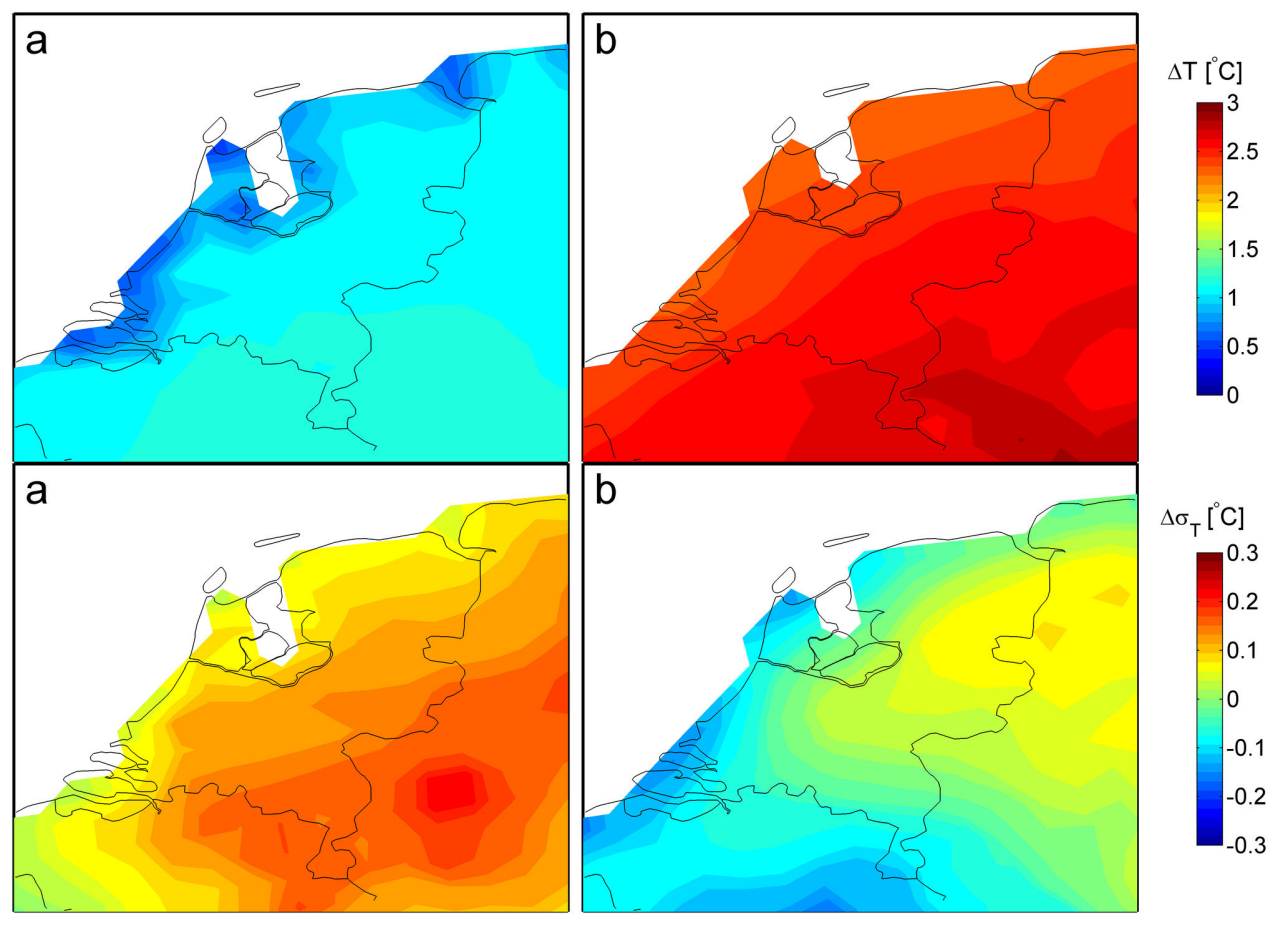
Changes in summer temperature. Changes (1975-1994 to 2031-2050) in mean summer (JJA) temperature (top panel) and standard deviation of summer mean monthly temperature (bottom) as projected by a) the KNMI model and b) the HC model.

**Figure 2:**
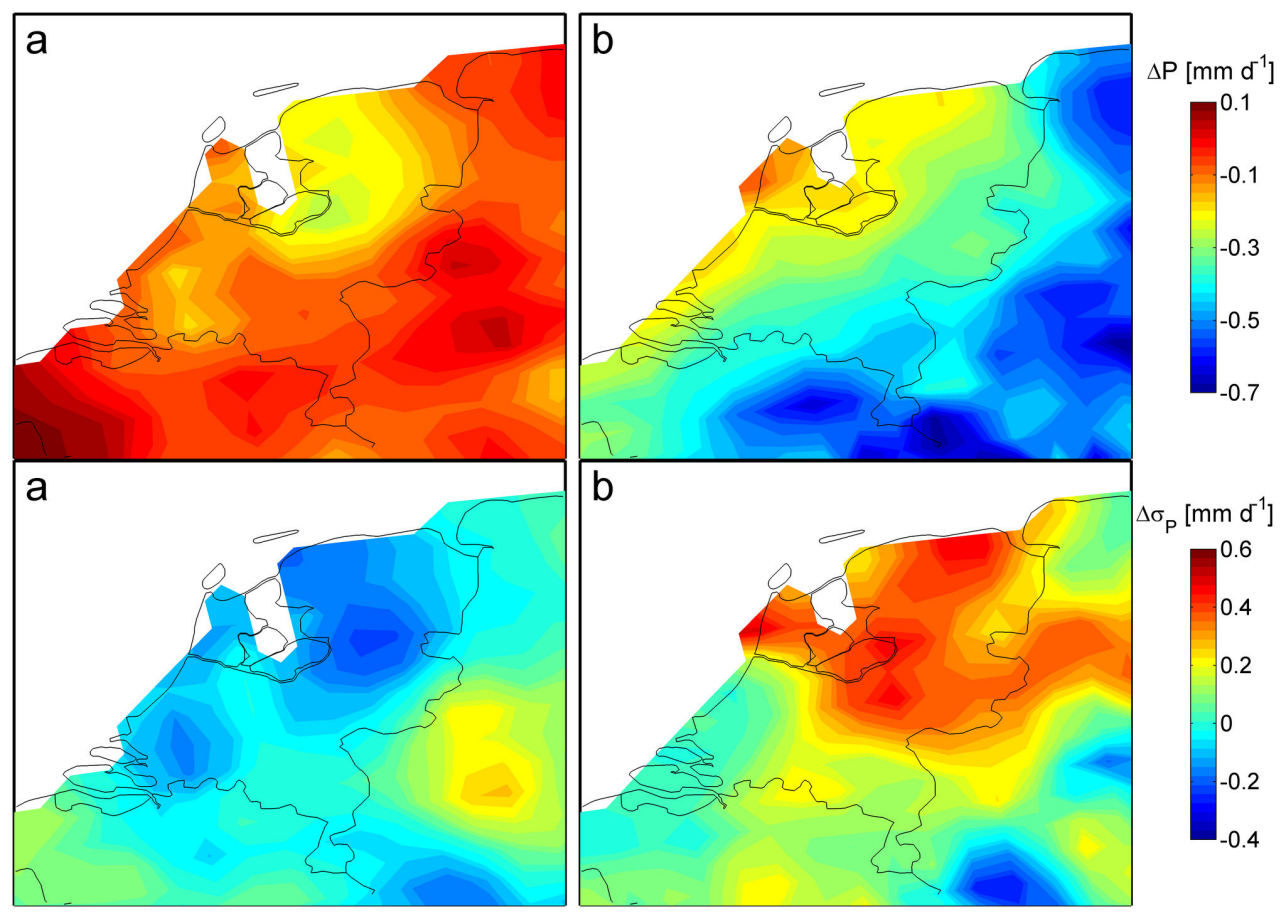
Changes in summer precipitation. Changes (1975-1994 to 2031-2050) in mean summer (JJA) precipitation (top panel) and in the standard deviation of mean monthly precipitation (bottom) as projected by a) the KNMI model and b) the HC model.

Figures 1 and 2 present the projected changes in the 20 year mean values (with standard deviation) of temperature and precipitation for the Netherlands - in the future period (2031-2050) relative to the baseline period (1975-1994) - as projected by the KNMI model and the HC model. Monthly mean changes of temperature, precipitation and relative humidity for April to September are shown in Table 1 (KNMI and HC). Both the KNMI and the HC models projected higher mean temperatures in both spring and summer with the largest increase in summer. The HC temperature increase was about 2 °C in spring and generally 0.7-1.5 °C larger than the increase projected by the KNMI model (Table 1). Both models projected a reduction in temperature variability in April and May (data not shown).

**Table 1:** Monthly mean changes in temperature (° C), precipitation (mm/day) and relative humidity (%) as projected for the Netherlands by the KNMI model and the HC model for 2031-2050 relative to 1975-1994.

Weather variable	Calendar month					
*KNMI*	*April*	*May*	*June*	*July*	*August*	*September*
Temperature (°C)	1.2	0.7	1.1	0.9	1.2	0.6
Precipitation (mm/day)	0.0	-0.5	-0.4	0.0	-0.1	1.1
Relative humidity (%)	0.1	-0.2	-0.6	-0.5	-0.2	1.0
*HC*						
Temperature (°C)	2.1	1.8	2.6	2.1	1.9	2.2
Precipitation (mm/day)	0.3	-0.1	0.3	-0.4	-0.6	-0.05
Relative humidity (%)	-0.8	-1.5	-1.7	-5.9	-5.1	-2.2

The amount of spring/summer precipitation (in mm/day) is generally projected to decrease, but the seasonal pattern is rather different in the two model projections (Figure 2 and Table 1). The KNMI model projects a decrease in precipitation in May and June, whereas the HC model projects the largest decrease to occur in July and August. For the KNMI model, the decrease in variability of mean summer precipitation (Figure 2) was dominated by a considerable decrease in variability in August. Both the KNMI and the HC models projected reductions in mean relative humidity. The HC model projected the largest reduction in July and August, associated with higher temperatures and reduced precipitation.

### Crop phenology

**Figure 3:**
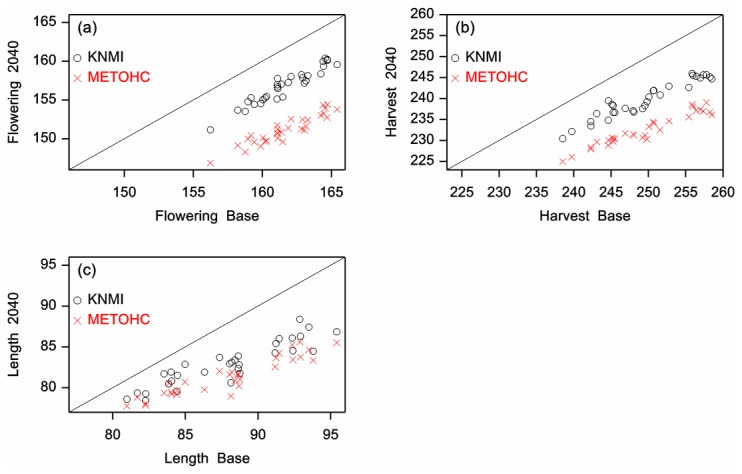
Estimated future flowering and full maturity dates of winter wheat. Estimated flowering (a) and full maturity dates (b) as well as difference between these two dates (length of grain filling period, c) as average of each grid (n=31) for winter wheat in the baseline period (1975-1994) and the future scenario period (2031-2050) using KNMI model data and HC climate model data. Dates are expressed as Julian dates.

Results for the simulated flowering and full maturity dates of wheat and maize for each of the KNMI and HC model projections, and shifts in these dates between the future and baseline scenario periods, are presented in Table 2 and Figure 3. The range of differences in both flowering and full maturity between the 31 grid cells are presented by the average, minimum, and maximum values (Table 2).

**Table 2:** Summary of project climate change effects on flowering date, full maturity date and concentrations of deoxynivalenol in winter wheat and maize grown in the Netherlands, based on KNMI and HC climate model data.

	*FD baseline* ^1^	*FD future* ^1^	*FD difference* ^1^	*MD baseline* ^1^	*MD future* ^1^	*MD difference* ^1^	*DON baseline* ^1,2^	*DON future* ^1^	*DON difference* ^1^
*Wheat*									
KNMI average	162	157	-5	250	240	-10	2564	2282	-282
Minimum	156	151	-6	239	230	-14	506	730	*
Maximum	165	160	-3	259	246	-5	5737	6361	*
HC average	162	151	-11	250	233	-17	2728	2634	-95
Minimum	156	147	-12	239	225	-22	560	914	*
Maximum	165	154	-9	259	239	-13	5957	12501	*
*Maize*									
KNMI average	215	208	-7	261	242	-19	188	33	-155
Minimum	205	198	-9	239	223	-28	5	1	*
Maximum	222	215	-4	278	251	-14	402	85	*
HC average	215	201	-14	261	225	-36	256	6	-250
Minimum	205	191	-17	239	210	-45	13	0	*
Maximum	222	208	-11	278	235	-29	545	34	*

^1^ FD: Flowering data (Ordinal date), MD: Full maturity date (Ordinal date), DON: deoxynivalenol concentration (in μg kg^-1^ , Diff: Difference between dates (in days), * Not calculated.

^2^ Note: differences in DON concentrations between KNMI and HC model data in the baseline period are due to differences in data on relative humidity; data on rainfall and temperature are identical since this series corresponded to the observed series.

In the future scenario period, flowering of winter wheat was estimated to be 3 to 6 days earlier with KNMI model data and 9 to 12 days earlier with HC data. Dates of full maturity were expected to be up to 2 weeks earlier with KNMI data, and up to 3 weeks earlier with HC data. Differences in wheat flowering and full maturity dates between baseline and future scenario period were significant (p<0.05) for each of the 31 grid cells as well as for the whole country (all grid cells), for both KNMI and HC model data. The length of the grain filling period was projected to be significantly shorter in the future period as compared to the baseline period for both climate model combinations. The overall difference (over 31 grid cells) was a reduction of 5 and 7 days for KNMI and HC model data, respectively.

As with wheat, both flowering and full maturity dates of maize were projected to occur earlier in the season in the future as compared to the baseline period (both climate model combinations). Flowering was expected to advance by 4 to 9 days using KNMI data and by 11 to 17 days for the HC model. Full maturity of maize advanced by 2 to 4 weeks with KNMI model data and by 4 to over 6 weeks with HC model data. This resulted in a shortened grain filling period of between 8 and 20 days with KNMI data (overall average 12 days) and between 15 and 31 days with HC data (overall average 22 days). All differences were significant (p<0.05), both on the grid cell level and the country level.

### Mycotoxin contamination

**Figure 4:**
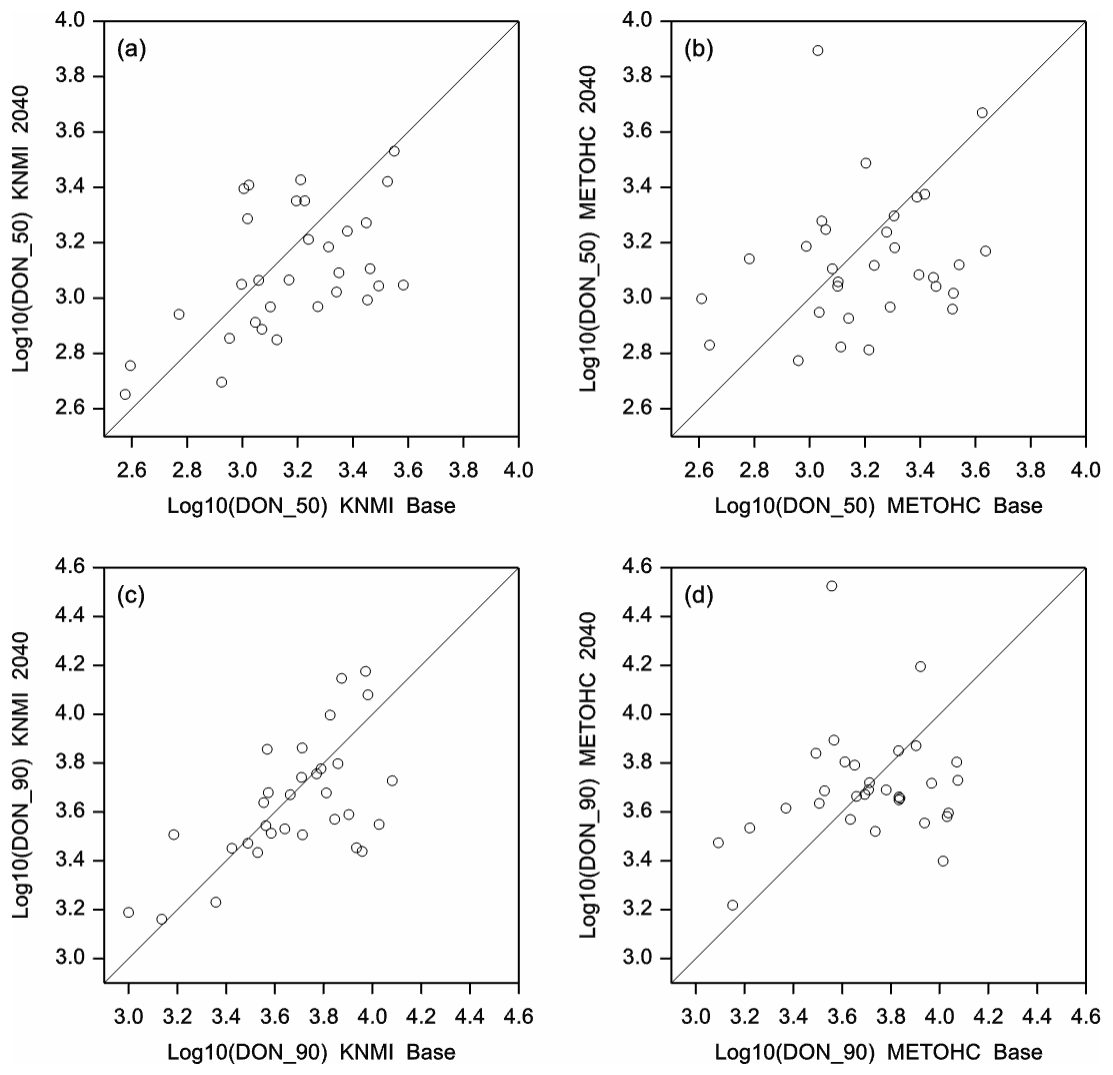
Estimated future DON concentration. Estimated DON concentration (log transformed, in μg kg^-1^) in the future scenario period (2031-2050) relative to the baseline period (1975-1994) using the KNMI model (left panels) and the HC (right panels) climate model data. DON contamination is expressed in both 50^th^ and 90^th^ percentile values.

In the baseline period, mean DON concentrations in wheat were estimated to range from about 500 to nearly 6000 μg kg^-1^ between the 31 grid cells in the Netherlands (Table 2). Changes in DON concentrations in the future relative to the baseline period were estimated to be relatively small on average; overall a non-significant decrease of nearly 300 μg kg^-1^ DON was projected. However, average differences in DON concentrations varied widely between the 31 grid cells in the Netherlands; one grid cell had a significant increase in DON concentration of 1807 μg kg^-1^ and - at the other end of the spectrum - a grid cell had a significant decrease in DON concentration of 3730 μg kg^-1^. In total, seven out of the 31 grid cells showed a significant increase in DON contamination, and eight grid cells showed a significant decrease. DON contamination did not change significantly for the remaining 16 grid cells. Plots of the 50^th^ and 90^th^ percentile values of DON concentrations in wheat in both the baseline and future scenarios also showed wide variation between the 31 grid cells (Figure 4). With HC model data, average DON concentrations were estimated to range between grid cells from nearly 600 μg kg^-1^ to nearly 6000 μg kg^-1^ in the baseline period. These values are slightly higher than obtained with KNMI model. Using HC model data, the projected shifts in DON contamination in the future relative to the baseline scenario also varied between the grid cells. One grid cell had a projected decrease of 3839 μg kg^-1^, and another one had a projected increase of 11059 μg kg^-1^; both differences were significant. In total, 10 grid cells showed a significant projected decrease in DON concentrations, and six grid cells had a significant increase. The overall average difference was estimated to be a slight and non-significant decrease (Table 2). With KNMI data, 27 out of the 31 grid cells had a mean DON concentration exceeding the EC limit of 1250 μg kg^-1^ for the presence of DON in unprocessed wheat [34], in both the baseline and the future scenario. With HC data, 30 grid cells had an estimated mean DON concentration exceeding this EC limit in the baseline situation, whereas in the future scenario, 27 grid cells had a DON concentration exceeding this limit.

DON contamination of maize in the future period was estimated to be lower than in the baseline period for all 31 grids cells and using both climate model combinations. With KNMI model data, DON concentrations in the baseline period ranged up^1^ to about 400 μg kg^-1^ between the 31 grid cells. For the future scenarios, projected concentrations will range between the various grid cells up kg^-1^ to 85 μg kg^-1^. For each of the 31 grid cells, DON contamination was projected to be reduced; this reduction was significant (p<0.05) for 26 grids cells. Future scenario calculations with HC model data showed a larger decrease than with KNMI data. The baseline period showed DON concentrations up to 545 μg kg^-1^. In the future scenario DON concentrations ranged up to 34 μg kg^-1^. The reduction in DON contamination was significant for 30 out of the 31 grid cells. On the country level, the reduction was significant for the HC model (p<0.05), but not for the KNMI.

## Discussion and Conclusion

Model results showed that timing of wheat flowering was projected to advance by 3 to 12 days depending on the region and climate change projection used. This would change the average flowering date of wheat grown in the Netherlands from mid June to early June, but still within the warm period of summer. Such changes in date of flowering are within the range found in other European studies for effects of projected changes of climate for the mid 21^st^ century [35,36]. Average date of full maturity of winter wheat was estimated to advance by 10 to 17 days on average, which ideally would improve the conditions for crop harvesting [37]. Shifts in DON contamination of wheat were not correlated to shifts in dates of flowering or full maturity dates of the crop; both higher and lower DON concentrations were found with earlier flowering and maturity in the future as compared to the baseline period (data not shown). In a preliminarily study, in which wheat flowering and maturity were assumed not to change, climate change was estimated to result into increased DON contamination in the Netherlands [38]. Obviously, shifts in the climate around flowering (and full maturity) play a role in DON contamination, rather than timing of these phenology stages themselves. From the various climatic variables in the model to predict DON in wheat, shifts in rainfall showed a clear relation to changes in DON contamination in the future versus the baseline period (data not shown). With climate change, rain in the period before flowering (May rain) was expected to decrease, and this decrease reduced DON contamination in the projected future climate. This result was expected as fungal infection and mycotoxin contamination are known to be higher with increased wetness before flowering [39]. The KNMI and the HC models have different timings of the projected reduction of spring/summer precipitation. The KNMI model projected the largest decrease in precipitation in late spring where it most effectively reduced DON contamination, whereas the HC model had the largest decrease during summer. These findings highlight the importance of using weather variables based on various climate model projections in order to assess uncertainty in the outcome of model predictions. Similar assessments of uncertainties across different climate change projections have been conducted for Europe based on GCMs and RCMs for ecosystem responses [40] and agroclimatic conditions [37]. These assessments have generally shown the same direction of change under climate change across large regions of Europe, but with different magnitude. Our results illustrate that there may be large uncertainties associated with projecting effects of climate change on phenomena, such as mycotoxin contamination, which are affected by subtle combinations of temperature and rainfall events.

In the predictive model for DON in maize, spore dispersal was assumed to be influenced by wind and rain. Subsequent germination is influenced by temperature and relative humidity. Fungal growth and toxin formation depend on air temperature and moisture content of the maize grain [33]. Future temperature during maize flowering and maturation was expected to increase by on average 1 °C and 2 °C using KNMI and HC model data, respectively. Higher air temperatures, when still below the optimum fungal temperatures, favour fungal germination, growth and toxin formation [41]. In case the frequency of temperatures above optimum fungal temperature increases, rates for germination, growth and toxin formation rapidly decline. An increase in weather temperature, therefore, can have two opposite effects. Average relative humidity decreased with around 0.5% and 5% (absolute values) with KNMI and HC data, respectively. Rainfall either was comparable in the future situation (KNMI data) or decreased with around 0.5 mm/day (HC data); this lower wetness may reduce fungal infection [42]. Apart from weather effects, the duration of the grain filling period decreased from on average 46 days in the baseline scenario to on average 34 days and 24 days with KNMI and HC model data, respectively. A shorter grain filling period resulted in reduced DON concentrations probably because time for fungal growth and toxin formation after infection was shorter. This analysis thus showed that the various climate change effects contributed in a different way to shifts in DON contamination in wheat and maize. For wheat, a decrease in rainfall prior to flowering was the main factor determining the difference in DON concentrations. For maize the decrease in DON contamination at harvest may be due to the shorter duration of the grain filling season as well as the reduction in relative humidity and the increase in temperatures above the optimum temperatures for germination, growth and toxin formation. However, more research is necessary to confirm these findings.

Both mycotoxin prediction models used in the present study are mostly based on weather data in specific stages of the crop phenology which are critical for fungal infection and mycotoxin formation. Other factors, such as the pre-crop and tillage practices, are known to affect mycotoxin contamination of wheat and maize as well, although to a lesser extent [43-46]. Effects of such agronomical practices were not considered in developing these models as their values were expected not to be known for the future. Farm management will to some extent be influenced by climate change [8]. In particular, climate change is expected to lead to an increasing cropping frequency with grain maize [47] which, depending on crop and soil management, may lead to higher risk of *Fusarium* spp. infestation. Our results, therefore, provide insight into trends of mycotoxin contamination of crops with climate change rather than accurate predictions. Another factor not included in the modelling of mycotoxin contamination of wheat and maize was the possible shift in the responsible fungal species. With climate change, species that currently mainly occur in Southern Europe, such as *F* *. verticilloides* in maize, may move northwards [48] and, consequently, other mycotoxins may be formed. This effect has not been incorporated in the present modelling approach, which focused primarily on predicting DON contamination in maize and wheat, assuming current species will be present in similar amounts in the future.

This study predicted climate change to result in a significantly lower DON contamination of maize grown in the Netherlands by the 2040s, when using HC model data, but showed no significant effects on the overall DON contamination of wheat (both climate model combinations) and maize, when using KNMI model data. These results do not coincide with expectations regarding the impact of climate change on (increased) mycotoxin contamination recently published in review papers [13-15]. The present study thus showed that it is worthwhile to quantify impacts of climate change effects on food safety using a modelling approach rather than making estimations based on qualitative considerations only. Also, it showed the relevance of accounting for shifts in crop phenology when making predictions of mycotoxin contamination of grains. Our results regarding expected climate change impact on DON contamination of wheat grown in the Netherlands in general confirm results of a previous study for north west Europe [21]. The latter study used a more general predictive model for DON contamination, and also pointed towards no or only a slight change – both increase and decrease – in DON contamination for grid cells relating to the Netherlands; these results were, however, not tested for significance. An identical quantitative modelling approach – as used in this study - could be followed to predict climate change impacts on other mycotoxins, cereal grain types, and/or countries such to establish insights into climate change impacts on mycotoxins on a broader scale. Moreover, the modelling approach applied here could be followed for assessing climate change impacts on other food safety hazards as well.

In conclusion, this study estimated impacts of climate change on crop phenology and mycotoxin contamination of wheat and maize cultivated in the Netherlands by the 2040s. On average (country level), future contamination of maize with DON was estimated to be reduced when using one climate model. For the other climate model and for wheat (both climate models) no significant changes were predicted. However, variation between regions was large, and in some areas a significant increase in DON concentration of wheat was predicted. Risk managers are, therefore, encouraged to continue monitoring of mycotoxins in cereal grains – in particular wheat - and to focus, as well as possible, on those regions with expected high contamination. We propose predictive modelling to be helpful in this respect.
